# Towards a Circular Economy in Electroless Pore-Plated Pd/PSS Composite Membranes: Pd Recovery and Porous Support Reuse

**DOI:** 10.3390/membranes16010028

**Published:** 2026-01-04

**Authors:** Alejandro J. Santos-Carballes, David Alique, Raúl Sanz, Arturo J. Vizcaíno, José A. Calles

**Affiliations:** 1 Chemical and Environmental Engineering Group (GIQA), Rey Juan Carlos University (URJC), c/Tulipán s/n, 28933 Mostoles, Spain; alejandro.santosc@urjc.es (A.J.S.-C.); david.alique@urjc.es (D.A.); raul.sanz@urjc.es (R.S.); 2 Instituto de Tecnologías para la Sostenibilidad (ITPS), Rey Juan Carlos University (URJC), c/Tulipán s/n, 28933 Mostoles, Spain

**Keywords:** pure hydrogen, palladium membrane, porous stainless steel, planar membrane, electroless plating, leaching, recycling, circular economy

## Abstract

The recycling of a planar composite Pd membrane over a porous stainless-steel support modified with a CeO_2_ interlayer (Pd/CeO_2_/PSS) was investigated using a leaching-based recycling strategy to recover palladium while maintaining the support’s structural integrity. The membrane was prepared by a continuous flowing electroless pore-plating method (cf-ELP-PP) previously developed by our group. A series of experiments was conducted to evaluate the effect of leaching conditions—temperature, acid concentration, and duration—on Pd extraction and support preservation. Nitric acid (HNO_3_) was used as the leaching agent, and the condition of 30 vol.% HNO_3_ at 35 °C for 24 h was found to enable complete Pd recovery with limited dissolution of metals from the support. The regenerated supports exhibited an Fe-Cr oxide layer and part of the CeO_2_ interface, allowing the elimination of cleaning and calcination steps in the membrane reprocessing workflow. A new Pd-CeO_2_ interfacial layer was applied, followed by Pd redeposition via cf-ELP-PP. The resulting recycled membrane exhibited a homogeneous and defect-free Pd layer, with hydrogen permeation performance comparable to that of membranes fabricated on fresh supports. These results demonstrate that Pd membranes can be successfully fabricated on recycled 316L stainless-steel substrates, supporting the viability of this approach for material reuse in membrane technology.

## 1. Introduction

The global energy transition, driven by the urgent need to reduce environmental and geopolitical risks associated with fossil fuel consumption, has intensified interest in hydrogen as a clean and flexible energy carrier [1,2,3]. Its ability to store energy from intermittent renewable sources and supply it in demand positions hydrogen as a key enabler of low-carbon technologies. However, many applications, particularly proton-exchange membrane fuel cells, require high-purity hydrogen to prevent catalyst poisoning and performance degradation [4].

Thermochemical hydrogen production methods, such as steam reforming or gasification of biomass-derived feedstock, offer significant efficiency advantages over water electrolysis. They provide integrated heat and mass flows, leverage existing industrial infrastructure, and generally incur lower capital and operating costs for large-scale hydrogen deployment [5,6,7,8,9,10]. However, hydrogen produced through these techniques does not meet the stringent purity requirements needed for certain applications.

To increase purity, several hydrogen physical separation technologies have been developed, including adsorption (pressure swing adsorption—PSA; temperature swing adsorption—TSA; and vacuum swing adsorption—VSA), cryogenic distillation, and membrane-based separation (polymeric, ceramic, and metallic membranes) [11]. Among them, dense metallic membranes have emerged as a promising solution due to their compactness, high selectivity, and ability to operate at elevated temperatures, making them ideal candidates for seamless integration within thermochemical hydrogen production systems [6,7].

A wide range of metals has been explored for the fabrication of this type of membrane, including nickel, vanadium, niobium and tantalum [12,13,14,15,16]. Yet, membranes based on palladium (Pd) or its alloys remain the most widely used, due to their superior hydrogen permeability, exceptional selectivity, and mechanical stability under harsh operating conditions [17,18,19]. These membranes typically consist of a thin Pd layer deposited onto a porous support—either ceramic or metallic—and sometimes include interfacial layers (e.g., CeO_2_, TiO_2_, SiO_2_) to improve adhesion or prevent interdiffusion.

During long-term operation, the Pd layer can be degraded by exposure to high temperature, pressure fluctuations, and/or reactive species. This deterioration reduces hydrogen permeability or selectivity, even though the support often remains structurally intact. Given the high cost and environmental footprint of membrane supports, their recovery and reuse are essential for enhancing the sustainability of hydrogen purification systems [20,21].

Pd recovery is routine in the automotive sector, where it is leached from spent catalytic converters using strong acidic or oxidizing agents (HCl, HNO_3_, Cl_2_ or H_2_O_2_) [22]. Unfortunately, these aggressive treatments typically destroy the support matrix, preventing its reuse. Promising methods have been developed for selectively removing Pd from ceramic-supported membranes (e.g., α-Al_2_O_3_) while preserving support integrity [23,24]. In the case of metal-supported Pd membranes, there is a lack of systematic research on Pd removal and the recovery of reusable supports. However, recent studies have shown promising corrosion resistance of 316L stainless steel in nitric acid environments due to the formation of a stable passive oxide layer and chromium enrichment [25]. This suggests that nitric acid leaching could effectively remove Pd while leaving the steel support virtually unaffected.

Given the strategic importance of porous stainless-steel (PSS) supports in high-temperature membrane systems at industrial conditions, it is crucial to assess whether they can be effectively regenerated and reused after Pd removal. Therefore, this work aims to develop and evaluate a recycling strategy for such Pd/PSS membranes (even containing interfacial layers), focusing on the compatibility of nitric acid-based treatments with support preservation. The study addresses explicitly (i) the efficiency of Pd removal using nitric acid; (ii) the resulting surface morphology and porosity of the stainless-steel support; (iii) the structural integrity of interfacial layers (e.g., iron/chromium oxides and Pd-CeO_2_); and (iv) the hydrogen permeation performance of the regenerated membranes.

## 2. Materials and Methods

### 2.1. Fabrication of the Original Pd/CeO_2_/PSS Composite Membranes

**Figure 1:**
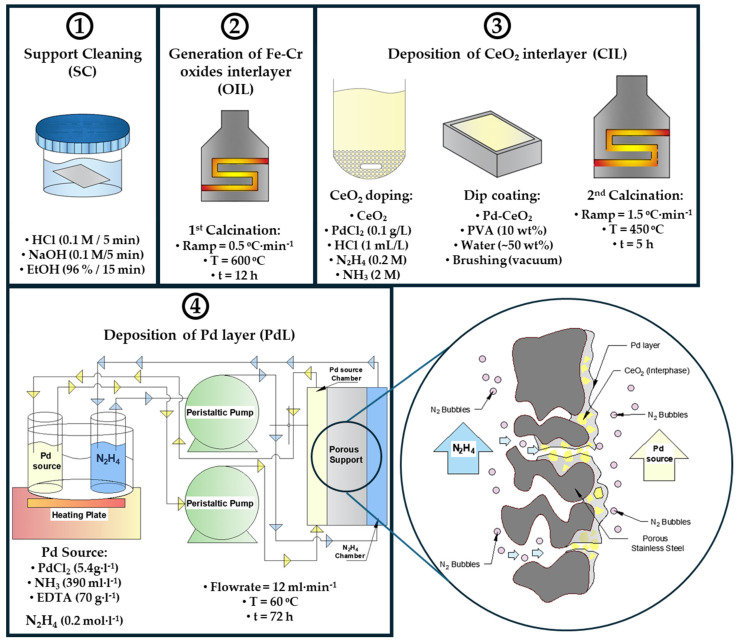
Pd-membrane fabrication process by cf-ELP-PP.

Planar Pd-based composite membranes were synthesized using the continuous flowing electroless pore-plating (cf-ELP-PP) method previously developed in our group [26]. Porous AISI 316L stainless steel (PSS) planar supports (90 × 60 × 1 mm, Mott Metallurgical Corp., Farmington, CT, USA) with 0.1 μm media grade were employed. The supports were laser-cut from commercial 200 × 300 mm foils, generating a densified 5 mm sealing frame to ensure proper sealing during both synthesis and permeation; the porous area was 4 × 10^3^ mm^2^. The synthesis of a membrane involved the sequential procedure schematized in Figure 1, as follows:

(1) Support cleaning (SC): Support was sequentially cleaned by ultrasonic baths (60 °C), using 250 mL of each solution: NaOH (0.1 M, 98% wt; Scharlau, Barcelona, Spain, CAS: 1310-73-2), HCl (0.1 M, 37% vol, Scharlau, Barcelona, Spain, CAS: 7647-01-0), and ethanol (96% vol, Scharlau, Barcelona, Spain, CAS: 64-17-5), with intermediate rinsing using deionized water between steps.

(2) Generation of Fe-Cr oxides interlayer (OIL): Cleaned support was calcined in air at 600 °C for 12 h (Nabetherm LT 60/11, Lilienthal, Germany, 0.5 °C·min^−1^) to form a thin Fe-Cr oxide barrier layer, preventing intermetallic diffusion between the support and the Pd layer.

(3) Deposition of Pd-CeO_2_ interlayer (CIL): Surface modification of the support was performed by depositing CeO_2_ particles (99.5%, Alfa Aesar, Haverhill, MA, USA, CAS: 1306-38-3), pre-activated with Pd nuclei (Pd-CeO_2_) as described elsewhere [18]. A viscous suspension was prepared by dispersing the dried Pd-CeO_2_ particles into a 10 wt% polyvinyl alcohol (PVA) solution (Sigma Aldrich, San Luis, MO, USA, M_W_ 8000–10,000, 80% hydrolyzed). Deionized water was gradually added to the mixture to adjust the final slurry composition to approximately 50 wt% total water content, yielding a suspension with adequate viscosity and adhesion for deposition. The resulting slurry was applied to the PSS support surface by vacuum-assisted brushing to promote particle infiltration into the porous structure and generate the Pd-CeO_2_ interlayer. The coated supports were dried (110 °C, overnight) and calcined at 450 °C for 5 h to remove organic material and consolidate the Pd-CeO_2_ interlayer.

(4) Deposition of the Pd layer by cf-ELP-PP (PdL): The selective Pd layer was grown on the modified support via cf-ELP-PP inside a custom-built deposition chamber equipped with ethylene-propylene copolymer seals and transparent windows for visual monitoring. A metal source solution was prepared containing 5.4 g·L^−1^ PdCl_2_ (98% vol., Sigma Aldrich, CAS: 7647-10-1), 70 g·L^−1^ EDTA (Scharlau, CAS: 10378-23-1) and 390 mL·L^−1^ NH_3_ (32%, Scharlau, CAS: 1336-21-6). A separate reducing solution was prepared with 0.2 M N_2_H_4_ (Scharlau, CAS: 7803-57-8). Both solutions were continuously recirculated on opposite sides of the porous support, pumped tangentially to the support surface at 12 mL·min^−1^ (Masterflex Ismatec peristaltic pump, Avantor, Barrington, IL, USA) at 60 °C for approximately 72 h. The continuous flow configuration provided constant homogenization of reactant concentrations across the membrane surface, preventing the formation of local concentration gradients. In addition, both recirculating streams were fed into the deposition cell from the bottom and discharged from the top, facilitating the upward displacement and efficient removal of nitrogen gas bubbles generated as by-products of the Pd reduction reaction. This configuration minimizes bubble accumulation on the surface of the growing film, thereby reducing the formation of defects. However, complete removal of gas entrapment within the porous structure cannot be guaranteed, although no critical defects derived from trapped bubbles were detected in the final membranes. Diverse mitigation strategies such as continuous or pulsed overpressure on plating solutions can be considered, although their implementation could interfere with plating chemistry and deposition kinetics. In addition, no evidence of bath cross-contamination was observed during the 72 h cf-ELP-PP process. Both solutions maintained their original appearance, while ICP-AES analysis of the reducing solution after prolonged circulation revealed only trace Fe (<20 ppm) and no detectable Pd.

After deposition, the membrane was rinsed with deionized water, leak-tested under helium (up to 3 bar), immersed in ethanol, and dried at 110 °C overnight, yielding a highly selective and defect-free Pd composite membrane.

In this study, two membranes were prepared following the above-described fabrication procedure: one of them (designated as M1) was used in the study for the selection of the conditions for selective Pd layer removal, and the other one (M2) was used to apply the support recovery protocol under the selected conditions and to fabricate a new membrane using the recovered support. The amount of material incorporated onto the support at each fabrication step was determined by weight gain using an electronic balance (Kern & Sohn ABS-4, Balingen, Germany) with an accuracy of ±0.1 mg. This measurement allowed the estimation of the gravimetric thickness of the Pd layer, assuming homogeneous metal incorporation onto the surface of modified PSS supports. The morphology and surface composition of the membranes were examined by scanning electron microscopy coupled with energy-dispersive X-ray spectroscopy (SEM-EDX, Thermo Fisher Scientific Phenom XL, Eindhoven, The Netherlands). Surface roughness was also evaluated using the Phenom 3D Roughness Reconstruction (3DRR) module, which reconstructs topography from backscattered electron images via a shape-from-shading algorithm. Semi-quantitative Ra and Rz values were determined from multiple fields of view and reported as mean ± standard deviation only for comparison purposes. The crystallographic structure of the support at the different stages of the membrane fabrication process was analyzed by X-ray diffraction (XRD, Malvern Panalytical Empyrean, Malvern, UK) using Cu Kα radiation. The patterns were recorded in the range 2θ = 20–70 ° with an increment step of 0.017° and a collection time of 2 s per step. The obtained diffractograms were compared with reference data from the JCPDS database to identify crystalline phases.

### 2.2. Pd Layer Removal via Nitric Acid Leaching and Preparation of a New Membrane from the Recovered Support

The removal of the Pd layer from Pd/CeO_2_/PSS membranes by HNO_3_ leaching was studied under different operating conditions, evaluating the support integrity. For this purpose, the M1 membrane was cut into small fragments (0.5 g), which were immersed in HNO_3_ solutions under continuous stirring (300 rpm), controlling temperature by means of a thermostatic bath. The tests were carried out varying the nitric acid concentration (15–65 vol.%), temperature (35–50 °C) and leaching time (0.25–24 h), using 100 mL of HNO_3_ solution per gram of membrane. After each treatment, samples were thoroughly washed with deionized water and dried at 110 °C for 24 h.

In order to analyze the evolution of Pd recovery and metal extraction from the support over leaching time under the different operating conditions tested, liquid aliquots were collected during the experiments. Metal concentrations (Pd, Fe, Cr, Ni and Ce) in the liquid samples were determined by inductively coupled plasma atomic emission spectroscopy (ICP-AES, Varian Vista AX Pro, Palo Alto, CA, USA). Before measurement, 0.5 mL of leaching solution was diluted in 4.5 mL of distilled water. On the other hand, the morphology and surface composition of the supports used in leaching tests were analyzed by SEM-EDX (Thermo Fisher Scientific Phenom XL, Eindhoven, The Netherlands), and the surface roughness was estimated by the Phenom 3D Roughness Reconstruction (3DRR) module. To evaluate the presence of residual Pd on the surface, five points were examined in five different regions of each sample. Pd contents higher than 0.5 wt.% were considered as a detectable amount based on EDX sensitivity.

Once leaching conditions were selected, membrane M2, previously tested under various permeation conditions and considered to be at the end of its useful life—after a stress test at 550 °C and 3 bar that caused irreversible damage to the Pd layer while maintaining the structural integrity of the support—was subjected to the established leaching protocol to remove the Pd layer while preserving the porous support for reuse. The recovered PSS support was subsequently reprocessed to synthesize a new membrane (r-M2) by adapting the standard fabrication procedure described in Section 2.1.

### 2.3. Permeation Tests

**Figure 2:**
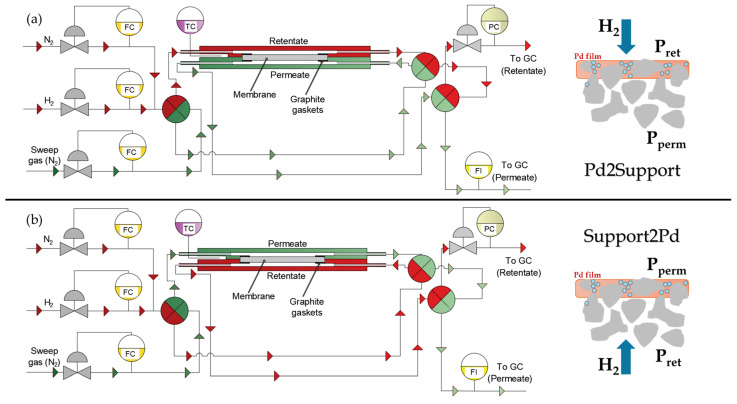
Process diagram of the permeation facility in different configurations: (**a**) Pd2Support; (**b**) Support2Pd.

The permeation behavior of both original M2 and recycled r-M2 membranes at different stages of their fabrication processes was measured to evaluate the success of the recycling procedure and the regeneration of membrane performance. Tests under diverse permeation conditions were performed in the experimental facility, schematically represented in Figure 2.

The membrane was installed in a custom-designed permeation cell constructed from 316L stainless steel. Graphite gaskets were used to ensure gas-tight separation between the retentate and permeate compartments, which was confirmed by leak test prior to each experiment. The cell was externally heated using flexible heating blankets. At the same time, the membrane temperature was precisely controlled by a Eurotherm EPC3016 controller (Eurotherm, Worthing, UK), which was connected to a K-type thermocouple positioned near the membrane surface.

Hydrogen and nitrogen flow rates were controlled using EL-FLOW mass flow controllers (Bronkhorst High-Tech, Ruurlo, The Netherlands, model F-201CV-RGD-11-V, maximum capacity: 400 Nml·min^−1^). The system allowed for the introduction of either pure gases or H_2_/N_2_ mixtures, while permeation direction could be reversed via three four-way valves. Therefore, permeate fluxes can pass first through the Pd-film and then the bulk porous media (Pd2Support configuration) or vice versa (Support2Pd configuration). These two contrary configurations were implemented to assess the influence of eventual mass transfer resistances in the modified porous media, as well as to demonstrate the mechanical integrity of the membranes even in the case of working under unfavorable conditions with the generation of tensile stress on the Pd-film. Permeate flow rates were monitored using mass flow meters (Bronkhorst High-Tech, Ruurlo, The Netherlands, model F-111B-RGD-11-V, maximum capacity: 250 NmL·min^−1^). For low flow rates (<5 NmL·min^−1^), a Horiba SF-1U/2U bubble flow meter (Horiba Ltd., Kyoto, Japan) was employed to enhance measurement accuracy and reproducibility.

The retentate pressure was maintained between 0.2 and 2 bar using an EL-PRESS back-pressure regulator (Bronkhorst High-Tech, Ruurlo, The Netherlands, model P-702CV-AGD-11-V), while the permeate side was kept at atmospheric pressure without a sweep gas. Permeation experiments were conducted at temperatures ranging from 350 to 475 °C.

Gas compositions in both retentate and permeate streams were analyzed using a gas chromatograph (Varian CP-4900, Palo Alto, CA, USA) equipped with a thermal conductivity detector (TCD) and two analytical columns (Molsieve 5A and PoraPLOT-Q). Prior to each permeation test, membrane integrity was verified by pressurizing the retentate side with helium at 3 bar and confirming the absence of gas flow on the permeate side.

## 3. Results and Discussion

### 3.1. Characterization of the Original Pd/CeO_2_/PSS Membranes

The two membranes (M1 and M2) prepared following the fabrication procedure described in Section 2.1 were characterized by weighing the membranes to analyze weight gain at different stages of the entire process and by scanning electron microscopy with energy-dispersive X-ray spectroscopy (SEM-EDX) to analyze their elemental composition.

To define the composition of the original membranes before being subjected to recycling, the masses per unit membrane area of surface of the Fe-Cr oxides formed in the OIL stage, the Pd-CeO_2_ interlayer deposited in the CIL stage, and the Pd layer deposited in the PdL stage were experimentally determined from weight-gain measurements. The results are displayed in Table 1, together with the estimated average thickness of the Pd layer. The reported values correlate quite well with those previously published [26] for membrane fabrication using the cf-ELP-PP method employed in this study. Moreover, the relative standard deviations (RSD) of the resulting properties remain below 10%, indicating reasonable reproducibility.

**Figure 3:**
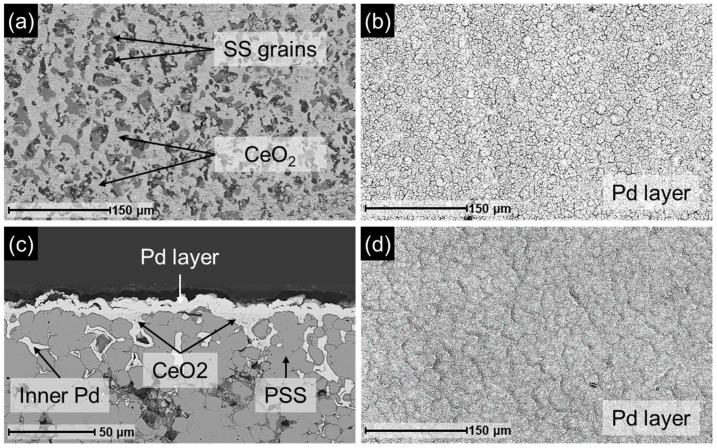
SEM images of original Pd/CeO_2_/PSS membranes at different fabrication steps: (**a**) M1 support after CIL stage; (**b**) M1 final top morphology after cf-ELP-PP; (**c**) M1 cross-section; (**d**) M2 final top morphology after cf-ELP-PP.

Prior to conducting leaching tests, it is crucial to assess both the external and internal morphology of the membranes, as their structural features may influence the Pd removal process from the support. For this purpose, the prepared membranes were characterized by SEM, and the results are presented in Figure 3.

Figure 3a displays the surface image of the M1 membrane support obtained after the CIL stage, prior to the deposition of the Pd layer. The support is partially covered by the Pd-CeO_2_ interlayer, while the grains of the calcined PSS support remain exposed. EDX analysis revealed an oxygen content exceeding 10 wt.% on these grains, indicating the presence of a mixed oxide layer, primarily composed of iron and chromium oxides [27]. The surface morphology of membrane M1 is shown in Figure 3b, where the Pd layer appears as a homogeneous and continuous film, free of surface defects that could compromise membrane selectivity, as it was corroborated by the absence of permeate when nitrogen was fed to the retentate side up to pressures of around 3 bar. A cross-sectional view of this membrane, shown in Figure 3c, reveals that Pd has infiltrated the porous structure of the support, anchoring the metallic layer and extending beyond the Pd-CeO_2_ interlayer at several locations, consistent with observations reported in previous studies [26]. Finally, Figure 3d illustrates the surface morphology of the M2 membrane, which also exhibits a homogeneous, continuous, and defect-free Pd layer. This morphology closely resembles that of membrane M1, as expected given the similar characteristics reported for both membranes in Table 1.

### 3.2. Effect of Temperature in Leaching Treatment for Pd Release from Pd/CeO_2_/PSS Membranes

The objective of this study is to identify suitable operating conditions that enable the complete removal of Pd from Pd/CeO_2_/PSS membranes while preserving the structural integrity of the support, thereby allowing the subsequent fabrication of a new membrane. To this end, leaching tests were initially performed on the M1 membrane using an acid solution containing 30 vol.% HNO_3_ at different temperatures (35, 40, and 50 °C). These experiments were conducted over 24 h, monitoring the evolution of metal extraction from the membrane.

**Figure 4:**
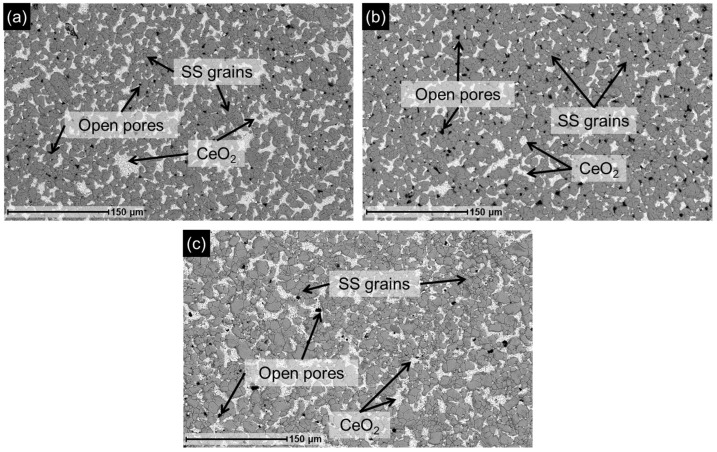
Membrane (M1) SEM images after leaching for 24 h at constant 30 vol.% HNO_3_ and different temperatures: (**a**) 35 °C; (**b**) 40 °C; and (**c**) 50 °C.

Given that the aim of this work is to obtain a functional membrane using a recycled support, it is essential to ensure that the surface morphology of the recovered support is suitable for Pd membrane fabrication. Therefore, the support obtained after the leaching treatment at the different temperatures was characterized by SEM, and representative surface images are shown in Figure 4.

After leaching, the surface exhibited numerous open pores and randomly distributed CeO_2_ regions, while the metallic grains of the support remained in conditions similar to those observed in Figure 3a. In fact, all three analyzed surfaces displayed comparable morphological features. Additionally, elemental surface analysis by EDX was performed on these samples, and the results are summarized in Table 2. This analysis confirmed the absence of Pd on the surface (<0.5 wt.%). At the same time, the oxygen content remained above 10 wt.%, indicating that the Fe-Cr mixed oxide layer persisted on the support surface after leaching, although it may have undergone partial dissolution by acid exposure and subsequent regeneration under nitric acid conditions [25]. However, the varying amounts of Ce detected in each sample at the end of the leaching process suggest random variability in the detachment of CeO_2_ particles, which results in open pores on the surface, consistent with their random distribution across the treated supports observed in the images.

**Figure 5:**
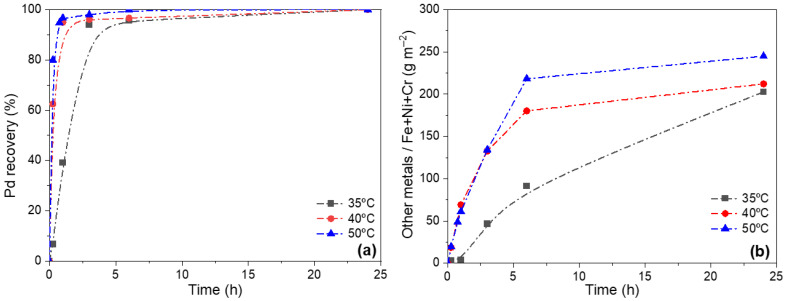
Evolution of leaching tests using 30 vol.% HNO_3_ at different temperatures: (**a**) Pd recovery from the membrane; (**b**) extraction of other metals from the support (g m^−2^).

The absence of detectable Pd after 24 h indicates that complete Pd removal from the surface was achieved under these leaching conditions, ensuring complete recovery of Pd in the acid solution. However, to further investigate the dynamics of metal extraction, the concentration of metals in the leachate was monitored over time using ICP-AES. This allowed for the estimation of both Pd recovery and the extent of metal leaching from the support throughout the treatment. As shown in Figure 5a, Pd recovery follows an asymptotic trend, with faster kinetics observed at higher temperatures. Complete Pd removal was achieved after 24 h at all tested temperatures, confirming the effectiveness of the leaching process under these conditions. Regarding the leaching of other metals from the support components, it is noteworthy that no Ce was detected in the acidic leachate due to the low solubility of CeO_2_ in HNO_3_ under the applied leaching conditions. Its partial detachment from the membrane surface is likely due to mechanical dislodgement or weakening of interfacial adhesion, rather than chemical dissolution. This detachment may result from the degradation of adjacent layers (such as Fe-Cr oxides) or from the loss of structural continuity following Pd removal. Consequently, open pores are formed on the surface, consistent with the random distribution of CeO_2_ particles across the treated support. Figure 5b shows that the total concentration of metals leached from the support increased with temperature. The cumulative extraction of Fe, Cr and Ni was lowest at 35 °C (202.6 g·m^−2^), whereas higher values were observed at 40 °C and 50 °C. This quantitative evidence supports reduced degradation at 35 °C, despite SEM analysis revealing no significant morphological differences among supports treated at different temperatures.

Since complete Pd removal was achieved within a reasonable timeframe (24 h) at all three evaluated temperatures, the recycling process was continued at 35 °C to minimize support degradation while ensuring full Pd recovery.

### 3.3. Effect of HNO_3_ Concentration in Leaching Treatment for Pd Release from Pd/CeO_2_/PSS Membranes

Following the evaluation of temperature effects on support degradation at a fixed acid concentration, the influence of nitric acid concentration was investigated at four levels: 15, 30, 45, and 65 vol.%. As in the previous experiments, leaching treatments were conducted over a 24 h period, analyzing both the surface morphology and composition of the recovered supports, as well as the concentration of metals in the leachate.

**Figure 6:**
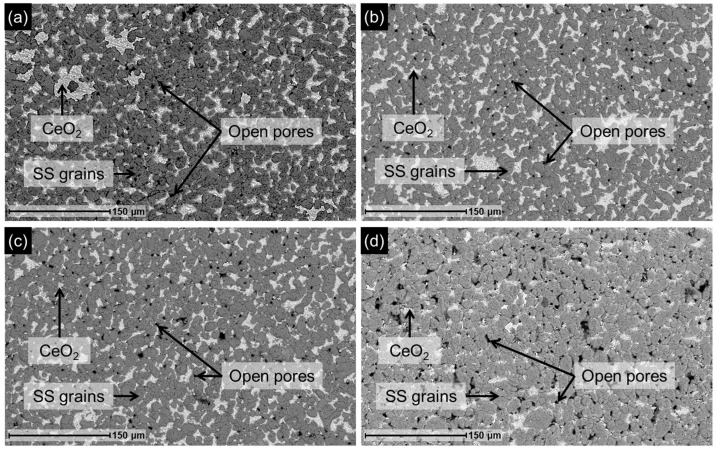
SEM images of M1 membrane after leaching for 24 h at constant temperature (35 °C) and different HNO_3_ concentrations: (**a**) 15 vol.%; (**b**) 30 vol.%; (**c**) 45 vol.%; (**d**) 65 vol.%.

The supports obtained after the 24 h leaching treatment at the different acid concentrations were characterized by SEM. Representative surface images are shown in Figure 6, revealing morphologies similar to those observed in Figure 4. In all cases, regions where Pd and portions of the CeO_2_ interlayer were removed are visible, exposing the underlying support and generating numerous open pores where CeO_2_ was present before detachment during the leaching process.

However, EDX analysis of the surface elemental composition, summarized in Table 3, reveals significant differences between samples treated at varying HNO_3_ concentrations. While no Pd was detected on the surface of membranes treated with 30 vol.% HNO_3_ or higher—indicating complete Pd removal—an average Pd content of 0.8 wt.% was measured across different regions in the sample treated with 15 vol.% HNO_3_, confirming the presence of residual Pd. The Fe-Cr mixed oxide layer was present on the surface in all cases, since the oxygen content remained above 10 wt.%. Again, the differences in Ce content observed among the samples at the end of the leaching process are consistent with a non-uniform detachment of CeO_2_ particles, leading to the random formation of open pores.

**Figure 7:**
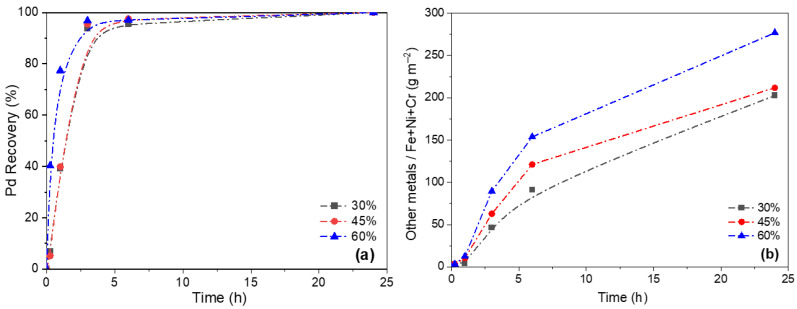
Evolution of leaching tests at 35 °C using different HNO_3_ concentrations: (**a**) Pd recovery from the membrane; (**b**) extraction of other metals from the support (g m^−2^).

Figure 7 illustrates the kinetics of metals removal from the membrane, with Figure 7a showing a rapid Pd extraction during the first 3 h for acid concentrations of 30, 45 and 65 vol.%, followed by a slower phase that reaches completion around 24 h. Regarding the leaching of metals associated with support degradation, Figure 7b shows that higher acid concentrations led to greater metal dissolution. These results confirm that recycling is feasible at HNO_3_ concentrations of 30 vol% or higher. However, a 30 vol.% HNO_3_ concentration ensures total Pd recovery while minimizing loss of structural metals from the support, thereby reducing degradation and preserving the original material.

Based on these findings, operating conditions of 30 vol.% HNO_3_ at 35 °C for 24 h are identified as appropriate for the recycling of Pd-based membranes supported on PSS.

### 3.4. Process for the Manufacture of a New Membrane with a Recycled Support

**Figure 8:**
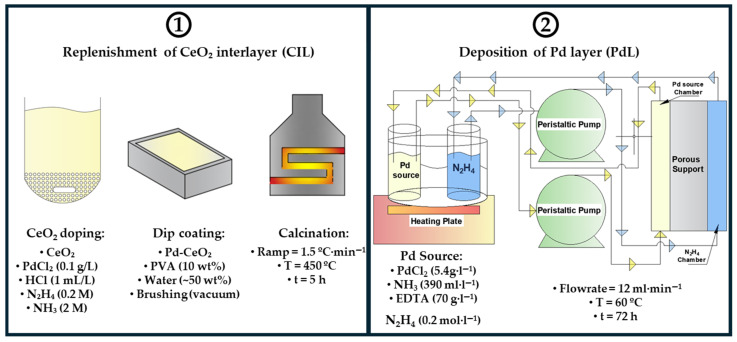
Pd-membrane fabrication process by cf-ELP-PP adapted to the use recycled supports.

Once the appropriate conditions for Pd removal were established, it was necessary to adapt the cf-ELP-PP membrane fabrication process to the specific characteristics of the regenerated support, which differs from the fresh one in several key aspects. The Fe-Cr mixed oxide layer is present after leaching, meaning that the initial calcination (OIL step) required for fresh supports is no longer necessary. Additionally, the standard cleaning step (SC), typically involving an HCl wash, should be omitted for recycled supports, as the acid could dissolve the oxide layer and necessitate a new calcination step, thereby increasing energy consumption [28]. On the other hand, portions of the CeO_2_ interlayer and its Pd doping—essential for proper Pd layer growth—are partially removed during leaching. As a result, the regenerated support must be recoated with Pd-CeO_2_ (CIL stage) to reseal the exposed pores and restore the doped interfacial layer required to initiate Pd nucleation during the selective layer generation by cf-ELP-PP (PdL step). A schematic representation of the adapted Pd/CeO_2_/PSS membranes fabrication process using recycled supports is provided in Figure 8.

To assess the viability of the proposed recycling and reprocessing strategy for PSS-supported Pd membranes, the recycling protocol was applied to the M2 membrane under the selected leaching conditions (30 vol.% HNO_3_ at 35 °C for 24 h). The recovered support was then used to fabricate a new membrane, referred to as r-M2, following the adapted synthesis procedure described above.

#### 3.4.1. Characterization of the Recovered Membrane Support

**Figure 9:**
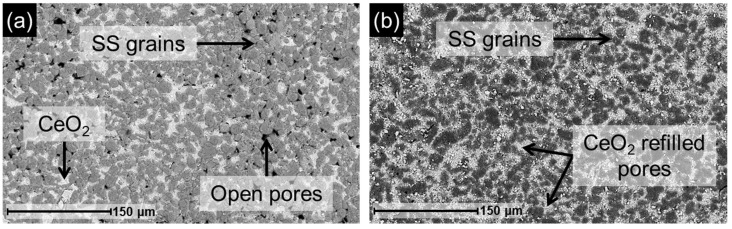
SEM images of the recovered support from M2 membrane after: (**a**) leaching, (**b**) CIL stage.

The morphology of the recovered support from the original M2 membrane was examined both before and after the regeneration of the CeO_2_ interlayer. The corresponding SEM surface images are presented in Figure 9, illustrating the evolution of the support throughout the reprocessing step. Following the leaching treatment, the surface morphology of the regenerated support is shown in Figure 9a, and exhibits features similar to those previously observed in Figure 6b for the M1 membrane support recovered under the same operating conditions. At this stage, Pd is no longer detectable on the surface (i.e., below 0.5 wt.%), while a substantial presence of surface oxides is evident. EDX analysis explicitly performed on the stainless-steel grains revealed an oxygen content of 27.3 wt.%, confirming the presence of the Fe-Cr mixed oxide layer even after the leaching process. These results are consistent with the findings discussed in Section 3.2 and Section 3.3. In terms of metal extraction, both M2 and M1 membranes exhibited comparable behavior, with total metal removal values of 222.6 g·m^−2^ and 202.6 g·m^−2^, respectively. After reapplying the Pd-CeO_2_ interfacial layer (Figure 9b), the surface appears to have resealed the open pores generated during leaching. The resulting morphology closely resembles that of the fresh support after the CIL stage, as shown in Figure 3a.

In addition, XRD analysis (Figure S1) confirmed the stability of the 316L stainless-steel support throughout the process. After the SC stage, the diffractogram exhibited only intense peaks corresponding to the austenitic phase (γ-Fe). Following oxidation (OIL stage), additional low-intensity peaks of Cr_1_._3_Fe_0_._7_O_3_ were detected, and after interlayer deposition (CIL stage), CeO_2_ peaks appeared with considerable intensity. After leaching, the recovered support displayed γ-Fe and Cr_1_._3_Fe_0_._7_O_3_ peaks, together with CeO_2_ peaks of significantly lower intensity, thus suggesting partial removal of this phase during acid treatment, in agreement with previous morphological and chemical observations. No peak shifts or additional phases were detected, confirming that the crystallographic integrity of the support was maintained after recycling.

**Figure 10:**
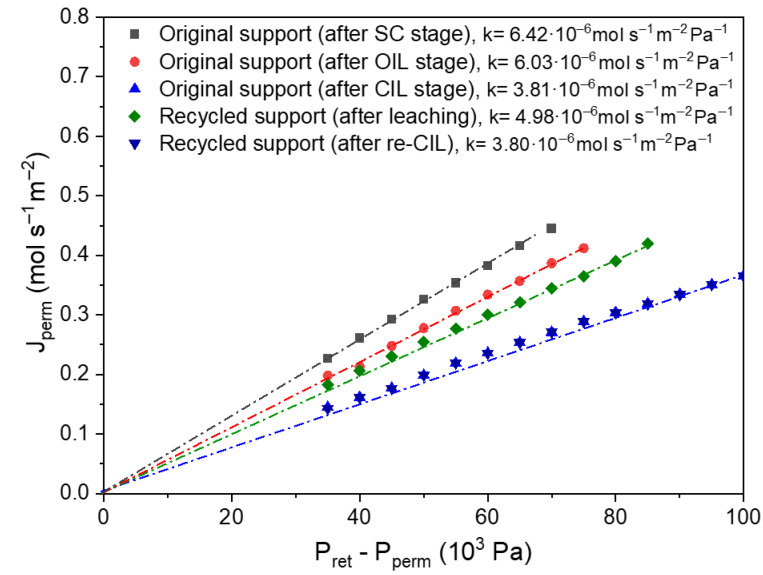
Nitrogen permeability of original and recycled supports at room temperature.

At this point, it is essential to verify that the internal structure of the support has not undergone significant degradation due to acid exposure. To assess this, nitrogen permeation measurements were performed at room temperature and compared with those of the fresh support at different stages (SC, OIL and CIL stages) of membrane synthesis. As shown in Figure 10**,** the nitrogen permeance of the support just after Pd extraction was 4.98 × 10^−6^ mol·m^−2^·s^−1^·Pa^−1^, an intermediate value between that of the fresh support after calcination (OIL stage), where the Fe-Cr layer has been formed, and the fresh support with the Pd-CeO_2_ interface (CIL stage). This result indicates that the support has only partially lost its surface CeO_2_ interface. Upon replenishment of the new Pd-CeO_2_ interfacial layer, where a mass gain of 4.1 g·m^−2^ was recorded, the nitrogen permeance of the support further decreases to 3.80 × 10^−6^ mol·m^−2^·s^−1^·Pa^−1^, which perfectly fits with that of the fresh support with the Pd-CeO_2_ interface, indicating that the newly applied interface effectively sealed the pores generated during the leaching process.

To reinforce this argument, a topographical roughness analysis of the surfaces before and after leaching was carried out by evaluating ten different profilometric sections obtained from SEM images, from which an average value and standard deviation were calculated. These values are semi-quantitative and intended only for comparative interpretation. For the original support after the SC stage, an average roughness (Ra) of 0.802 ± 0.094 μm was obtained, with a mean roughness depth (Rz) of 3.30 ± 0.42 μm. This roughness is significantly reduced after the CIL stage (Ra = 0.256 ± 0.036 μm; Rz = 0.84 ± 0.12 μm), confirming that the CeO_2_ layer effectively smooths the external surface of the porous substrates. After the leaching treatment to recover the support from spent membranes, the surface appearance shown in Figure 9a results in a roughness increase, yielding Ra = 0.673 ± 0.088 μm and Rz = 2.31 ± 0.27 μm, in agreement with the higher N_2_ permeation. These new values lie between those of PSS supports after the SC and CIL fabrication stages, which is consistent with a partial removal of ceria during the process. Finally, after introducing a new CeO_2_ layer, the roughness decreases again up to Ra = 0.260 ± 0.026 μm and Rz = 0.97 ± 0.12 μm, very similar to the values obtained while preparing a fresh membrane.

Given that both the surface morphology and nitrogen permeance of the recycled support closely match those of the original M2 membrane support, it can be concluded that the regenerated substrate has successfully recovered the essential characteristics required for Pd membrane growth by cf-ELP-PP, maintaining its structural integrity. Notably, this is achieved with a lower Pd-CeO_2_ loading than with a fresh support, highlighting the efficiency of the recycling strategy.

#### 3.4.2. Characterization and Performance of the Recycled Membrane

**Figure 11:**
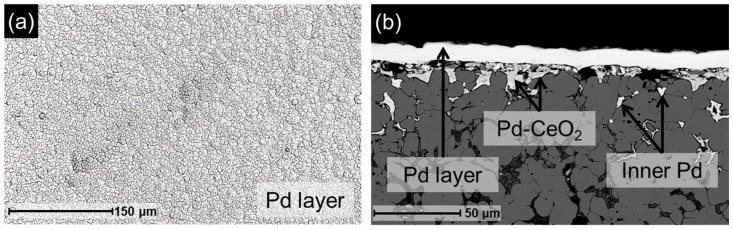
SEM images of the new r-M2 membrane prepared from a recycled support: (**a**) top morphology; (**b**) cross-section.

After replenishing the Pd-CeO_2_ interfacial layer, Pd was redeposited onto the regenerated support using the cf-ELP-PP method. The resulting membrane, designated as r-M2, was characterized by SEM, with representative images shown in Figure 11. The surface exhibits a continuous and homogeneous Pd layer, free of visible defects, which is essential for hydrogen selectivity. As shown in Figure 11a, the surface morphology closely resembles that of the original M2 membrane (Figure 3d). Cross-sectional analysis (Figure 11b) reveals a structure consistent with previously discussed results, including significant Pd infiltration into the porous support and the formation of a uniform Pd layer. Direct thickness measurements taken at 25 points on the cross-section yield an average value of 9.9 ± 0.9 µm for the new Pd layer, in agreement with the estimated gravimetric thickness of 10 µm. In addition, this value is comparable to the 8.5 µm measured for the fresh M2 membrane (Table 1) and to those previously published for similar membranes [26].

**Figure 12:**
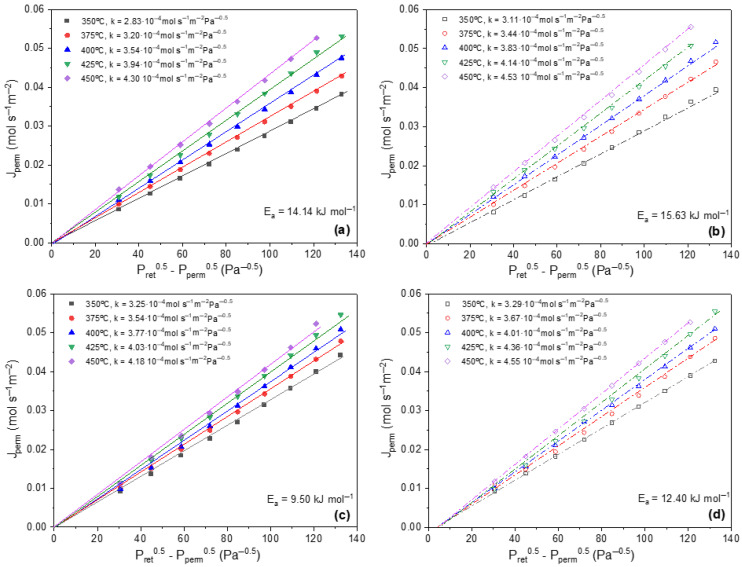
Hydrogen permeation for original and recycled membranes at contrary permeate flow direction: (**a**) M2, Pd2Support; (**b**) M2, Support2Pd; (**c**) r-M2, Pd2Support; (**d**) r-M2, Support2Pd.

Finally, permeation tests were conducted on the r-M2 membrane and compared with those of the original M2 membrane. In both cases, complete selectivity was verified by the absence of detectable permeate flow when 200 mL·min^−1^ of pure N_2_ was fed into the system at a pressure differential of up to 3 bar. This corresponds to an ideal hydrogen separation factor of ≥10,000, based on the minimum detection limit of the flowmeter (1 mL·h^−1^). Afterwards, permeation experiments using pure H_2_ were performed, and the results are presented in Figure 12.

Both membranes exhibit increased permeation when the flow direction is reversed from Pd2Support to Support2Pd configuration, a behavior previously reported for membranes fabricated via the ELP-PP process [18]. Additionally, when fitting the flow data to Sievert’s law, a slight deviation from the origin is observed in the Support2Pd direction. This deviation occurs despite the linear behavior of the data, and is attributed to additional transport resistance when hydrogen crosses the porous support before reaching the Pd layer, which introduces uncertainty in the effective partial pressure just on the retentate Pd-surface during Sieverts’ law fitting. However, these origin offsets are reduced with respect to those reported for tubular PSS-supported membranes prepared by ELP-PP, as already observed in our recent work [26]. This effect can be ascribed to the lower wall thickness and average porosity of planar supports, combined with the continuous flowing plating process, which ensures uniform pore sealing and homogeneous Pd deposition. The activation energy values obtained for both membranes fall within the range reported for membranes with similar structural characteristics, as summarized in Table 4.

Considering the similarity in cross-sectional structure and surface morphology with previously reported membranes [26], along with the comparable hydrogen permeation performance, it can be concluded that the proposed recycling strategy for 316L stainless-steel supports enables the fabrication of Pd membranes on recycled substrates while preserving both the structural integrity and functional properties of the final membrane.

## 4. Conclusions

This study demonstrates the feasibility of recovering and reusing Pd/CeO_2_/PSS membrane supports through a controlled leaching process. Nitric acid (HNO_3_) was shown to be effective for extracting the Pd layer. A systematic evaluation of leaching parameters—temperature, acid concentration, and duration—allowed the identification of suitable conditions for Pd recovery while limiting structural alteration of the support. Specifically, leaching at 35 °C with 30 vol.% HNO_3_ for 24 h enabled complete Pd extraction while preserving the structural integrity of the support, with minimal metal loss (31.4 mg·g^−1^ membrane).

The regenerated supports exhibited the Fe-Cr oxide layer and part of the CeO_2_ interface, eliminating the need for additional cleaning and calcination steps during membrane reprocessing. Replenishment of the Pd-CeO_2_ interfacial layer successfully restored the surface morphology and sealed the pores generated during leaching. Subsequent Pd deposition yielded a membrane with a homogeneous and defect-free surface, which was comparable in thickness (9.9 µm) and structure to membranes fabricated on fresh supports.

Hydrogen permeation tests confirmed that the recycled membrane exhibits a performance equivalent to that of the original membrane, including complete H_2_ selectivity and permeation behavior (3.77 × 10^−4^ mol·Pa^−0.5^·s^−1^·m^−2^). These findings validate the proposed recycling strategy as a viable and efficient approach for fabricating Pd membranes on reused 316L stainless-steel supports, contributing to material sustainability and process optimization in membrane technology.

This method can also be applied to common Pd alloys containing Ag, Cu or Ni, as these elements are more soluble in HNO_3_ than Pd. In contrast, Au is inert to nitric acid, so Pd-Au alloys would require additional treatment. Minor adjustments in operating conditions may be needed to account for differences in dissolution kinetics.

## Figures and Tables

**Table 1:** Characteristics of original Pd/CeO_2_/PSS membranes fabricated by cf-ELP-PP.

	M1	M2	[26]	RSD (%)
Fe-Cr oxides layer (g·m^−2^)	96.9	86.5	89.1	6.0
Pd-CeO_2_ (g·m^−2^)	9.2	8.1	7.9	8.3
Pd (g·m^−2^)	95.2	102.2	110.6	7.5
Pd gravimetric thickness (µm)	7.9	8.5	9.2	7.6

**Table 2:** EDX elemental analysis (wt.%) of the membrane surface (M1) after leaching at different temperatures.

Element	Leaching Temperature		
	35 °C	40 °C	50 °C
O	17.3 ± 1.2	16.9 ± 0.5	15.3 ± 1.3
Cr	13.9 ± 1.7	17.5 ± 0.4	17.3 ± 2.4
Fe	37.6 ± 5.6	50.5 ± 0.8	50.2 ± 3.5
Ni	5.0 ± 0.6	6.3 ± 0.2	6.8 ± 0.5
Pd	n.d.	n.d.	n.d.
Ce	25.8 ± 6.7	8.7 ± 1.6	10.2 ± 5.9

n.d.: not detected.

**Table 3:** EDX elemental analysis (wt.%) of the membrane surface (M1) after leaching at different concentrations.

Element	HNO_3_ Concentration (vol.%)			
	15%	30%	45%	65%
O	13.4 ± 1.3	17.3 ± 1.2	21.7 ± 2.2	17.0 ± 0.5
Cr	18.8 ± 1.5	13.9 ± 1.7	21.0 ± 3.1	17.5 ± 0.4
Fe	49.4 ± 0.4	37.6 ± 5.6	50.2 ± 1.6	50.6 ± 0.9
Ni	7.6 ± 0.5	5.0 ± 0.6	7.0 ± 0.4	6.3 ± 0.2
Pd	0.8 ± 0.2	n.d.	n.d.	n.d.
Ce	10.0 ± 3.0	25.8 ± 6.7	15.5 ± 15.0	8.5 ± 1.8

n.d.: not detected.

**Table 4:** Main characteristics and performance comparison with other Pd membranes from the literature.

Geometry	Support	Interlayer	Selective Layer	Thickness (µm)	T (°C)	∆P (kPa)	k (mol s^−1^ m^−2^ Pa^−0.5^)	E_a_ (kJ mol^−1^)	α_H2/N2_	Ref.
Planar	Al_2_O_3_	-	Pd-Ag	3.7	550	101.3	3.53 × 10^−6^	8.6	>1000	[29]
Tubular	Hastelloy X	Bohemite	Pd-Ag	6.8	500	100	4.47 × 10^−4 a^	6.53	512	[30]
Planar	PSS	WO_3_	Pd-Ag	12	500	900	2.06 × 10^−8 a^	14.7	>1000	[31]
Tubular	PSS	SAPO-34	Pd	9	450	100	0.71 × 10^−6 a^	17.1	866	[32]
Tubular	PSS	Pd-TiO_2_	Pd	9.7	400	200	2.97 × 10^−4^	14.9	>10,000	[33]
Tubular	PSS	Pd-CeO_2_	Pd	9.1	400	100	6.26 × 10^−4^	13.1	>10,000	[18]
Planar	PSS	Pd-CeO_2_	Pd	9.2	400	150	3.88 × 10^−4^ 4.28 × 10^−4^	8.2–9.8	>10,000	[26]
Planar	PSS	Pd-CeO_2_	Pd	8.5	400	150	3.57 × 10^−4^ 3.83 × 10^−4^	14.1–15	>10,000	This work
Planar	Recycled PSS	Pd-CeO_2_	Pd	9.9	400	150	3.77 × 10^−4^ 4.01 × 10^−4^	9.5–12.4	>10,000	This work

^a^ (mol s^−1^ m^−2^ Pa^−1^).

## Data Availability

The original data presented in this study are openly available in e-cienciadatos (https://edatos.consorciomadrono.es, accessed on 19 November 2025) at https://doi.org/10.21950/HK9GTB (accessed on 19 November 2025).

## Supplementary Materials

The following supporting information can be downloaded at https://www.mdpi.com/article/10.3390/membranes16010028/s1. Figure S1: XRD diffractograms of the M2 membrane support at different fabrication steps.
