# Supplementary material for: Towards a Circular Economy in Electroless Pore-Plated Pd/PSS Composite Membranes: Pd Recovery and Porous Support Reuse
Source: Membranes (Basel). 2026 Jan 4;16(1):28. doi: 10.3390/membranes16010028 (PMC12843777; doi:10.3390/membranes16010028)
Supplement: Supplementary file 1 [file membranes-16-00028-s001.zip › membranes-4036498-supplementary.pdf]

membranes

MDPI

Supplementary Material

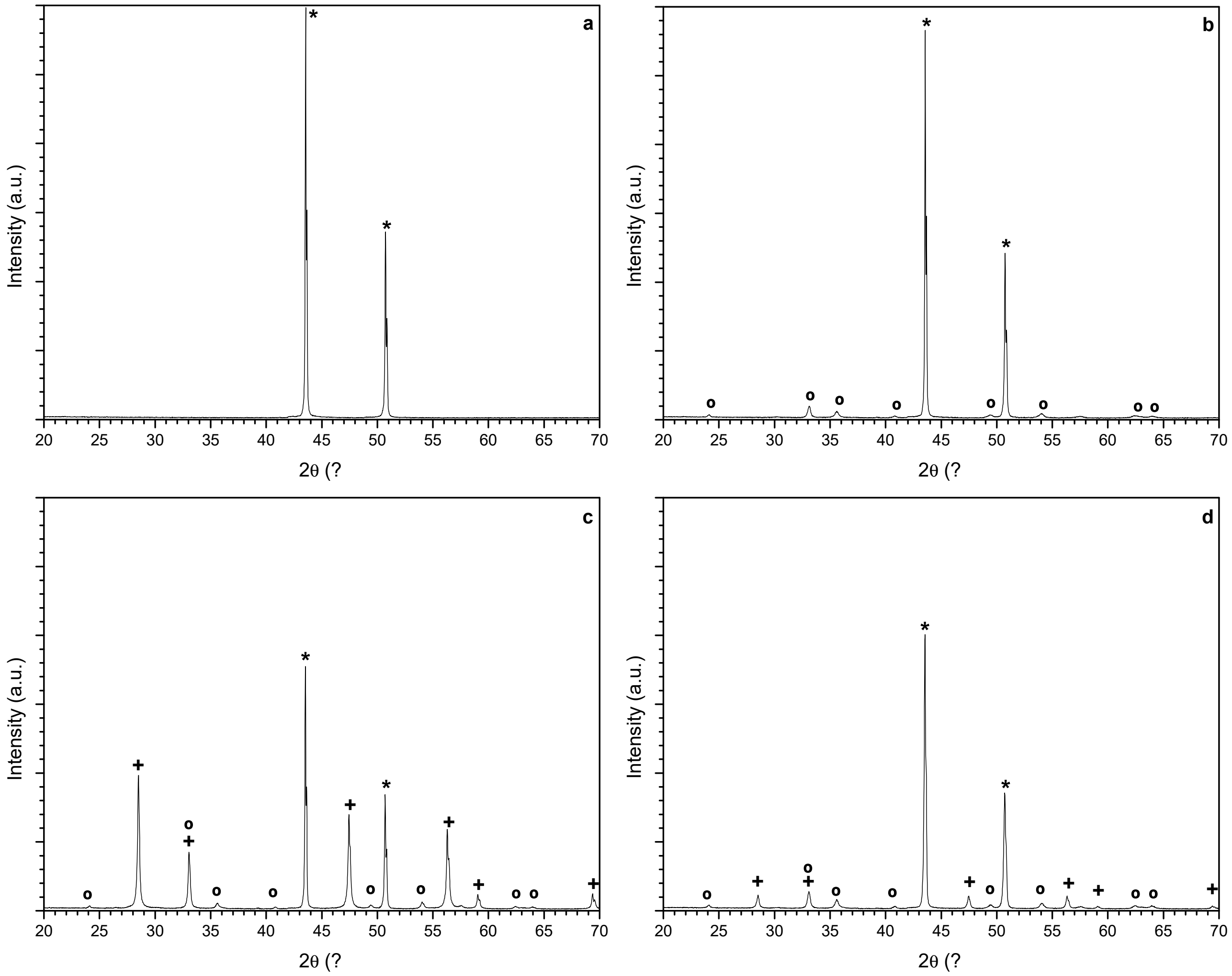

Figure S1. XRD diffractograms of the M2 membrane support at different fabrication steps: (a) Original support after SC stage; (b) Original support after OIL stage; (c) Original support after CIL stage; (d) Recycled support after leaching.

*: Austenite; o: $Cr_{1.3}Fe_{0.7}O_3$; +: CeO_2.
